# An fMRI investigation of empathic processing in boys with conduct problems and varying levels of callous-unemotional traits

**DOI:** 10.1016/j.nicl.2018.01.027

**Published:** 2018-02-28

**Authors:** Arjun Sethi, Elizabeth O'Nions, Eamon McCrory, Geoffrey Bird, Essi Viding

**Affiliations:** a Developmental Risk & Resilience Unit, Division of Psychology & Language Sciences, University College London, United Kingdom; b Department of Experimental Psychology, University of Oxford, United Kingdom

**Keywords:** Empathy, Affective introspection, Conduct problems, Callous-unemotional traits, Psychopathy, fMRI

## Abstract

The ability to empathise relies in part on using one's own affective experience to simulate the affective experience of others. This process is supported by a number of brain areas including the anterior insula (AI), anterior cingulate cortex (ACC), medial prefrontal cortex (mPFC), and the amygdala. Children with conduct problems (CP), and in particular those with high levels of callous-unemotional traits (CP/HCU) present with less empathy than their peers. They also show reduced neural response in areas supporting empathic processing when viewing other people in distress. The current study focused on identifying brain areas co-activated during affective introspection of: i) One's own emotions (‘Own emotion’); ii) Others' emotions (‘Other emotion’); and iii) One's feelings about others' emotions (‘Feel for other’) during fearful vs neutral scenarios in typically developing boys (TD; *n* = 31), boys with CP/HCU (*n* = 31), and boys with CP and low levels of CU (CP/LCU; *n* = 33). The conjunction analysis across conditions within the TD group revealed significant clusters of activation in the AI, ACC/mPFC, and occipital cortex. Conjunction analyses across conditions in the CP/HCU and CP/LCU groups did not identify these areas as significantly activated. However, follow-up analyses were not able to confirm statistically significant differences between groups across the whole network, and Bayes-factor analyses did not provide substantial support for either the null or alternate hypotheses. Post-hoc comparisons indicated that the lack of conjunction effects in the CP/HCU group may reflect reduced affective introspection in the ‘Other emotion’ and ‘Feel for other’ conditions, and by reduced affective introspection in the ‘Own emotion’ condition in the CP/LCU group. These findings provide limited and ultimately equivocal evidence for altered affective introspection regarding others in CP/HCU, and altered affective introspection for own emotions in CP/LCU, and highlight the need for further research to systematically investigate the precise nature of empathy deficits in children with CP.

## Highlights

• A core affective introspective network was detected in typically developing children
• This was active when introspecting about their emotions, another's emotions, or how they felt for another
• This network was not consistently detected in children with conduct problems
• Direct comparisons were however unable to detect significant differences in this network between groups
• Future work must carefully assess and compare deliberative and incidental socio-affective processing in these children

## 1 Introduction

Empathy is the capacity to understand and resonate with the affective experience of another (Singer and Lamm, 2009; Lockwood, 2016). Empathy plays a key role in inhibiting aggression and promoting prosocial behaviour (Eisenberg et al., 2005; Decety et al., 2016) and emerges early in development (Decety et al., 2016). Atypical empathic processing is thought to be a risk factor for the development of psychopathy (Blair et al., 2014). The ability of individuals with psychopathy to hurt and manipulate other people without concern for their welfare, suggests an atypical empathic/vicarious response to other people's distress (Viding and McCrory, 2015; Lockwood, 2016). Although it would be entirely inappropriate (and indeed erroneous) to diagnose children with psychopathy, a subgroup of children with conduct problems (CP) who also present with callous-unemotional (CU) traits present with atypical empathic responses to other people's suffering (Blair et al., 2014; Viding and McCrory, 2015). Such a pattern is similar to that seen in adults with psychopathy (Blair et al., 2014; Seara-Cardoso and Viding, 2015). Children with CP and high levels of CU traits (CP/HCU) are at a substantially increased risk of developing persistent antisocial behaviour compared with both typically developing (TD) peers and peers with CP and lower levels of CU traits (CP/HCU) (Frick et al., 2014; Viding and McCrory, 2015). There have been considerable efforts in recent years to understand the neurocognitive basis of empathy problems observed in these children (Blair et al., 2014; Viding and McCrory, 2015).

Empathic processing in typically developing children is supported by a number of brain regions that have been implicated in understanding and resonating with the affective experience of others (Singer and Lamm, 2009; Lockwood, 2016). Across both animal and human studies two brain areas, the anterior insula (AI) and anterior cingulate cortex (ACC), have been robustly associated with empathic processing/vicarious processing of emotions (Singer and Lamm, 2009; Lockwood, 2016). Other areas, such as inferior frontal gyrus and amygdala, have also been implicated in empathic processing (Singer and Lamm, 2009; Lockwood, 2016).

Neuroimaging studies have shown that children with CP, in particular those with CP/HCU, show atypical response to others' pain and distress in regions implicated in empathic processing (e.g. Lockwood et al., 2013; Marsh et al., 2013; Michalska et al., 2016; Yoder et al., 2016). For example, Marsh et al. (2013) reported reduced amygdala and ACC responses to photographs of pain-inducing injuries in children with CP, with those with HCU reporting the most pronounced differences compared with typically developing (TD) children. Lockwood et al. (2013) reported reduced AI and ACC responses to photographs of hands and feet in painful situations in boys with CP, with the degree of activation correlating negatively with the level of CU traits. Michalska et al. (2016) studied youth with CP as they viewed video clips of intentional and unintentional harm and reported that both CP and CU traits were negatively associated with AI and anterior midcingulate responses to intentional harm. Finally, Yoder et al. (2016) reporting on the same sample and task showed that HCU was associated with disrupted connectivity between ACC and AI and ACC and amygdala. They concluded that HCU is characterized by the disruption to cortical networks involved in detecting and appropriately responding to salient environmental cues, such as other people's distress.

This pattern of findings is in line with data from adults with high levels of psychopathic traits or diagnosed psychopathy. In several studies probing neural response to expressions of pain or distress, and other people's body parts experiencing pleasant or painful touch, adults with psychopathic traits/diagnosed psychopathy show lower AI activity (but see Decety et al., 2013 for a study reporting greater AI activity in incarcerated psychopaths in response to facial expressions of pain), and lower ACC response to these stimuli (e.g. Decety et al., 2013; Meffert et al., 2013; Seara-Cardoso et al., 2015, Seara-Cardoso et al., 2016). Interestingly, Meffert et al. (2013) report that when individuals with psychopathy are instructed to “empathise” whilst observing other people's hands receiving pleasant or painful touch, the group differences between neural responses in AI, ACC and IFG seen between individuals with psychopathy and typically developing controls are reduced, i.e. the neural response in those with psychopathy is no longer significantly lower than that which is seen in controls.

We wanted to further investigate the neural basis of empathy in children with CP/HCU, as contrasted with CP/LCU and TD children who were matched for age, SES and ability. We were particularly interested in isolating those brain areas that support processes thought to be critically involved in introspection about one's own emotions and thinking about others' emotions, which are thought to be involved in empathic responses to others (de Vignemont and Singer, 2006; Bird and Viding, 2014). We therefore sought to investigate regions co-activated during these processes. To achieve our aim we used a task that incorporated conditions requiring: (i) introspection about one's own feelings when picturing oneself in a fear-inducing situation; (ii) thinking about what another person is feeling when picturing him in a fear-inducing situation; and (iii) introspection about one's own feelings in response to hearing about another person in a fear-inducing situation. We reasoned that common brain areas activated across all of these conditions would be particularly important for resonating with and understanding others' emotions and using one's own affective state to guide these processes.

Our task enabled us to first identify those brain areas that are commonly activated during all these processes in typically developing (TD) children using conjunction analyses. Based on previous research we hypothesised that the AI and ACC should activate across all the task conditions. We then assessed whether such a network observed in TD children was also consistently activated by children in CP/HCU and CP/LCU groups across conditions. We further examined whether there were differences in activity in any of these core affective introspective regions specifically, by directly comparing groups across these conditions using a functional ROI approach informed by the previous conjunction analyses. We hypothesised that, compared to TD children, children with CP/HCU would show significantly reduced activation of AI and ACC. No directional hypothesis was made for CP/LCU children as previous work has primarily indicated that affective resonance/empathy deficits are more robustly associated with the children with CP/HCU rather than their peers with lower CU traits (Jones et al., 2010; Schwenck et al., 2012; but see Martin-Key et al., 2016), but this group was also compared with TD children on an exploratory basis.

## 2 Methods

### 2.1 Participants

Boys aged 11–16 years were recruited from the community via newspaper advertisements, and local mainstream and specialist provision schools. Screening questionnaires were administered to parents of 360 boys and teachers of 215 boys whose families expressed an interest in taking part and provided informed consent. The screening measures provided a research diagnosis of current conduct problems; dimensional assessment of CU traits; an overall screen for psychopathology; demographic data for group-matching purposes (i.e. socioeconomic status, parent-defined ethnicity, and handedness); and information regarding previous neurological or psychiatric diagnoses.

Current conduct disorder symptoms were assessed using the Child and Adolescent Symptom Inventory–4R (CASI-4R) Conduct Disorder (CASI-CD) subscale (Gadow and Sprafkin, 2009). CU traits were assessed using the Inventory of Callous-Unemotional Traits (ICU) (Essau et al., 2006). Both were scored by taking the highest ratings from either the parent or the teacher questionnaire for any given item (Piacentini et al., 1992). For the CASI-CD scale, inclusion in the conduct problem group required that the score met either parent or teacher severity cut-off (parent report: cut-off = 4+ [ages 10–12] and 3+ [ages 12–16]; teacher report: cut-off = 3+ [ages 10–12], 4+ [ages 12–14], and 6+ [ages 15–16]). These scores are associated with a clinical diagnosis of conduct disorder (Sprafkin and Gadow, 1998). Typically developing participants were required to score in the normal range for this measure, and below the cut-off for total difficulties on the Strengths and Difficulties Questionnaire (Goodman, 1997).

Automatic exclusion criteria for both conduct problems and typically developing groups included a previous diagnosis of any neurological or psychotic disorder, or current psychiatric medication. To recruit a representative group of children with conduct problems, common comorbidities (ADHD, generalized anxiety disorder [GAD], depression, and substance/alcohol abuse) were not used as exclusion criteria, but current parent-reported symptom counts were obtained during scanning sessions, so that their possible contribution to the findings could be systematically assessed.

On the basis of the screening information, one hundred participants took part in the fMRI scanning session. Participants were provided with a complete description of the study. Informed consent was obtained from parents and written assent from all participants. All aspects of the study were approved by the University College London Research Ethics Committee (Project ID number: 0622/001) and work was conducted in accordance with the Declaration of Helsinki.

Three participants (one with CP and two typically developing) withdrew from the session prior to collection of the task data due to poor tolerance of the scanner environment. Of the sample who completed scanning (66 participants with CP, 31 typically developing participants), data from 2 participants (both CP/HCU) were excluded due to image artifacts and poor registration. The remaining boys in the CP group were divided into HCU and LCU groups based on a median split of their scores on the ICU. All typically developing participants scored below the CP group median (42.24) on the ICU. Demographic and questionnaire data for participants are summarized in Table 1.

**Table 1:** Participant demographics.

	Mean (SD)						
Measure	TD	CP/LCU	CP/HCU	ANOVA	TD vs CP/LCU	TD vs CP/HCU	CP/LCU vs CP/HCU
Age	14.01 (1.759)	14.53 (1.579)	14.65 (1.411)	*F*(2, 92) = 1.45, *p* = 0.239	–	–	*–*
SES	2.81 (1.135)	2.64 (1.150)	3.06 (0.834)	*F*(2, 89) = 1.25, *p* = 0.293	*–*	*–*	*–*
IQ	100.97 (12.194)	101.44 (14.321)	97.10 (11.430)	*F*(2, 90) = 1.07, *p* = 0.349	*–*	*–*	*–*
Handedness (L/R)	4/27	3/30	4/27	*×*^2^(2, 92) < 0.01, *p* = 1.000			
Callous-unemotional traits	24.97 (6.750)	33.56 (7.538)	50.94 (6.870)	*F*(2, 92) = 108.64, *p* < 0.001	*p* < 0.001	*p* < 0.001	*p* < 0.001
Conduct problems (ASI)	0.23 (0.425)	3.18 (2.128)	5.60 (2.387)	*F*(2, 91) = 63.80, *p* < 0.001	*p* < 0.001	*p* < 0.001	*p* < 0.001
Conduct problems (SDQ)	1.19 (1.223)	4.00 (1.984)	6.64 (1.853)	*F*(2, 92) = 77.38, *p* < 0.001	*p* < 0.001	*p* < 0.001	*p* < 0.001
ADHD	12.34 (7.732)	23.28 (11.467)	24.68 (10.768)	*F*(2, 91) = 13.75, *p* < 0.001	*p* < 0.001	*p* < 0.001	*p* = 1.000
Generalized Anxiety Disorder	3.72 (1.932)	8.73 (5.082)	9.02 (3.779)	*F*(2, 91) = 18.55, *p* < 0.001	*p* < 0.001	*p* < 0.001	*p* = 0.001
Major Depressive Episode	3.19 (1.832)	6.40 (3.958)	6.95 (4.215)	*F*(2, 90) = 10.43, *p* < 0.001	*p* = 0.001	*p* < 0.001	*p* = 1.000
AUDIT	0.51 (1.467)	3.37 (5.931)	1.98 (2.737)	*F*(2, 92) = 4.26, *p* = 0.017	*p* = 0.013	*p* = 0.429	*p* = 0.477
DUDIT	0.13 (0.718)	3.21 (4.713)	2.00 (4.426)	*F*(2, 90) = 5.36, *p* = 0.006	*p* = 0.005	*p* = 0.165	*p* = 0.623

### 2.2 Psychometric and questionnaire measures

During the experimental session, participants completed the two-subtest version of the Wechsler Abbreviated Scale of Intelligence (Wechsler, 1999), the Alcohol Use Disorder Identification Test (Babor et al., 2001) and the Drug Use Disorder Identification Test (Berman et al., 2005) whilst parents completed the full CASI-4R (Sprafkin and Gadow, 1998). Between group analyses were conducted with and anxiety, depression and ADHD symptoms as covariates.

### 2.3 Experimental task

The task comprised three conditions, in which participants viewed fear-inducing or neutral scenes and heard a short audio description of a neutral or an emotionally charged situation, in which either they or another boy (described as being about their age and referred to as ‘J’) was the protagonist. After hearing about each situation, participants were asked to rate either (i) how they feel imagining themselves in the situation (‘Own’ condition), (ii) to imagine how ‘J’ feels in the situation (‘Other’ condition), or (iii) how they feel hearing about ‘J’ in the situation (‘Feel for other’ condition).

Participants were initially presented with a cue, which alerted them to whether the trial would ask them about how they feel (‘Own’ condition), how ‘J’ feels (‘Other’ condition), or how they feel hearing about ‘J (‘Feel for other’ condition) [duration = 3200 msec]. Next, they viewed a fear-inducing or neutral scene, and listened to a description of the scenario (e.g. ‘You’ fear condition: ‘You see a face in the window of an empty building’; ‘You’ neutral condition: ‘You see a boy riding a bike down the street’). The duration of visual stimuli presentation was 4950 msec.

Subsequently, participants were asked to rate their own or J's emotional state using a four point likert-scale. One end of the rating scale (green) was labelled as ‘not bad at all’, whilst the other (red) end was labelled ‘very bad’. For the ‘Own’ fear and ‘Own’ neutral conditions, the rating pertained to the participant's own emotional state (i.e. ‘How do **YOU** feel?’). For the “Other” condition, the question was ‘How does **J** feel?’, and for the “Feel for other” condition, the question was ‘How do **YOU** feel hearing about J?’. Once the participant had made their selection using a button box, the option that they had chosen was highlighted and remained visible on the screen until the end of the response window (3200 msec). After a 500 msec interval, the next trial began.

Experimental blocks consisted of 12 stimuli (two scenarios from each of the six conditions: (1) ‘Own’ fear, (2) ‘Other’ fear, (3) ‘Feel for other’ fear, (4) ‘Own’ neutral, (5) ‘Other’ neutral, (6) ‘Feel for other’ neutral. At the end of each experimental block, participants viewed a fixation cross for 14,000 msec. There were six experimental blocks in total, resulting in a task duration of 15 min 37 s. Within blocks, stimuli from the same condition were always presented in pairs. The order in which scenarios were presented within a condition across the task was generated randomly. The order in which scenario pairs for each condition were presented within an experimental-block was pseudo-randomised and determined from one of ten randomly selected run lists. These lists constrained the number of consecutive repetitions of any one emotion, such that there could be a maximum of four fearful or neutral scenarios presented in a row. Stimuli sets were balanced across groups and conditions, and each participant saw each scenario only once. See Supplementary materials for further information on task development.

The experimental task was administered using Cogent 2000 (Cogent 2000, Functional Imaging Lab/Institute of Cognitive Neuroscience, UCL, UK). Auditory vignettes were played via a Sony STR-DH510 digital AV control center (Sony, Basingstoke, UK) and MRI-compatible insert earphones (Sensimetrics Corporation, Malden, MA, USA). Noise attenuation was achieved through careful fitting and insertion of correctly sized silicone headphone tips, and custom made foam ear cushions adjusted to accommodate the participant's head.

### 2.4 Scanning

A Siemens Avanto 1.5-T MRI scanner (Siemens Medical, Erlangen, Germany) using a 32-channel birdcage head coil was used to acquire a 5.5-minute three-dimensional T1-weighted structural scan, and multislice T2*-weighted echo planar volumes with blood‑oxygen level-dependent contrast. The echo planar imaging sequence was designed to optimize signal detection and reduce dropout in the orbitofrontal cortex and amygdala (Weiskopf et al., 2006). Acquisition parameters were as follows: 42 2-mm slices acquired in an ascending trajectory with a 1-mm gap (voxel size = 3 × 3 × 2 mm); TE = 50 ms; slice repetition time = 87 msec, TR = 3654 msec; slice tilt = 25°±5° (TC); flip angle = 90°; field of view = 192 mm; phase oversampling = 12%. Functional data were acquired in a single run, with 265 volumes collected. Pre-scan normalised images were used in analyses.

### 2.5 fMRI preprocessing

Data were analysed using Statistical Parametric Mapping software (SPM version 8; Wellcome Trust Centre for Neuroimaging, UK). EPI volumes for each participant were realigned to their mean, before EPI and anatomical images were co-registered using the mean EPI and T1-weighted volumes. T1-weighted volumes were then segmented, with the resulting deformation fields used to normalise subjects' EPI volumes to the MNI template. EPI volumes were resampled to 1.5 mm^3^, before being smoothed with a Gaussian Full-Width Half-Maximum kernel of 8 mm^3^. Volumes with >0.5 mm motion or 1 degree of rotation were automatically detected with a custom script and manually checked for motion artefacts. Volumes with visible artefacts were replaced with the average of the preceding and succeeding volume, with a nuisance variable included in the design matrix to de-weight interpolated volumes.

### 2.6 fMRI analysis

Individual conditions (‘Own’ fear, ‘Other’ fear, ‘Feel for other’ fear, ‘Own’ neutral, ‘Other’ neutral, ‘Feel for other’ neutral, Fixation) were entered into the first level model as regressors of interest and convolved with the haemodynamic response function and its temporal derivative. Each trial onset was coded as the beginning of each vignette presentation, and ending after the decision phase of each trial. All data were high pass filtered at 128 Hz.

For inputting into second level analyses, individual contrasts were derived to specifically examine *affective* introspection within each condition (i.e. each fear condition – its respective neutral condition). To determine common regions implicated in affective introspection across these conditions, we performed conjunction analyses. Conjunction analyses were conducted separately for TDs, CP/HCU and CP/LCU to enable the examination of the neural substrates of affective introspection for each group, which was particularly critical for ascertaining that TD children engage brain areas typically deployed for affective introspection. Whole brain conjunction analyses were corrected at cluster level *p* = 0.05, and family-wise error was determined via 10,000 Monte-Carlo simulations with the AFNI programme 3dClustSim (http://afni.nimh.nih.gov/afni; voxel-wise *p* < 0.005, *k* = 638). Due to the small size of the AI, here significance in conjunction analyses was determined at voxelwise FWE *p* < 0.05 after small volume correction (SVC) using a mask derived from Deen et al. (2011). Significant clusters from the TD within-group analysis were deemed to form part of a core affective introspective network and were binarised for use as ROIs for later analyses. Raw averaged parameter estimates were extracted from these ROIs across conditions to test for group differences in overall affective introspection. To see if these differences were driven by any particular context, we also examined the parameter estimates from these ROIs for each condition separately.

Data were also analysed within a Bayesian framework using JASP (JASP Team, 2017), in order to examine the strength of the evidence in favour of the null and experimental hypotheses. Bayes Factors provide a ratio of the likelihood of the observed data under the null vs alternative hypothesis, whereas *p*-values examine the probability of the data given the null hypothesis and therefore cannot discriminate between evidence for the null and no evidence for either the null or alternative hypothesis (Dienes, 2015). This is particularly important for the present analyses, as this approach makes it possible to quantify the support for the null hypothesis between groups (i.e. to quantify evidence for a lack of group differences in any condition) as a follow up to similar or divergent results in the within group conjunction analyses. Bayes Factors (BF_10_) are reported below, where values approaching zero indicate that the data provide more evidence in favour of null hypothesis than the alternative hypothesis, a value of 1 indicates that the null and alternative hypotheses are equally likely given the data, and values above 1 indicate greater support for the alternative hypothesis. By convention values <1/3 and >3 are taken as evidence in favour of the null and alternative hypotheses, respectively, whilst values within these boundaries are judged to provide no evidence in favour of either the null or alternative hypotheses.

## 3 Results

### 3.1 Demographics

Groups were matched for age (*p* = 0.239), IQ (*p* = 0.349), SES (*p* = 0.293) and handedness (*p* = 1.000; Table 1). As expected, the groups differed significantly on measures of conduct problems and callous-unemotional traits (Table 1). For both measures, the CP/HCU and CP/LCU groups showed elevated scores compared to TDs (all *p* < 0.001), with the CP/HCU also having higher scores than the CP/LCU group (both *p* < 0.001; Table 1). We observed additional group differences in ADHD symptom severity scales with elevated scores in both CP groups compared to TDs (both: *p* < 0.001), but the CP/HCU and CP/LCU groups did not differ from each other (*p* = 1.000). We also observed group differences in depression and anxiety scores with CP/HCU and CP/LCU groups, again elevated compared to the TD group (all *p* < 0.001), but the CP groups did not differ from each other (both: *p* = 1.000; Table 1). There were also a significant effect of group on AUDIT (*p* = 0.017) and DUDIT (*p* = 0.006) scores, with elevated alcohol (*p* = 0.013) and substance use (*p* = 0.005) in the CP/LCU compared to the TDs group only (Table 1).

### 3.2 Behavioural

There was an effect of condition (*F*_*(2, 184)*_ = 9.70; *p* < 0.001) on ratings of fearful scenarios, which was driven by significantly higher ratings within the ‘Other’ emotion condition compared to ‘Own’ emotion (*p* = 0.006) and ‘Feel for Other’ emotion (*p* < 0.001; Table 2). There was no statistically significant effect of group on fear ratings across conditions (*F*_*(2, 92)*_ = 2.49; *p* = 0.088) and no significant interaction between group and condition (*F*_*(4, 92)*_ = 0.77; *p* = 0.545).

**Table 2:** Behavioural ratings for each fear condition.

	Mean (SD)			
Condition	TD	CP/LCU	CP/HCU	ANOVA
Own emotion	3.50 (0.385)	3.29 (0.469)	3.19 (0.678)	*F*(2, 92) = 2.80, *p* = 0.066
Other emotion	3.54 (0.333)	3.45 (0.375)	3.40 (0.380)	*F*(2, 92) = 1.30, *p* = 0.277
Feel for other	3.40 (0.459)	3.20 (0.595)	3.21 (0.509)	*F*(2, 92) = 1.57, *p* = 0.214

### 3.3 fMRI

#### 3.3.1 Conjunction analyses

**Fig. 1:**
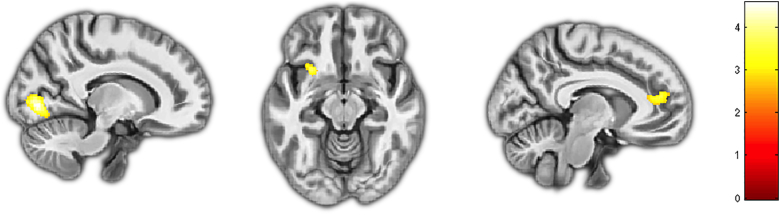
Conjunction analysis across ‘Own’ emotion, ‘Other’ emotion, and ‘Feel for Other’ conditions reveals a core affective introspective network comprising the AI, occipital lobe, and ACC/mPFC.

Conjunction analysis within the TD group across each condition (‘Own’ fear – neutral, ‘Other’ fear – neutral, ‘Feel for other’ fear - neutral) revealed significant clusters (*p* < 0.005, *k* > 638) within the occipital (*k* = 728; *x* = 14, *y* = −78, *z* = −1) lobe as well a cluster spanning the ACC and the medial prefrontal cortex (*k* = 670; *x* = −8, *y* = 44, *z* = 15; Fig. 1). After SVC, a significant conjunction effect within the AI was also detected (SVC *p* = 0.023; *k* = 130; *x* = −27, *y* = 14, *z* = −13). These clusters were not observed within the CP/HCU or CP/LCU groups, with no significant clusters observed within these regions even at more lenient statistical thresholds (*p < 0.01; k > 638)*.

#### 3.3.2 Group comparisons

We next tested whether there was a statistically significant between-group difference in neural activation in those areas implicated in the TD conjunction analysis. No statistically significant between-group differences were found in AI and ACC/MPFC in our ROI analyses (*p >* 0.05; Table 3). We did, however, observe a significant group effect within the occipital lobe (*F*_*(2, 92)*_ = 5.36, *p* = 0.006), with post hoc comparisons revealing significantly reduced activity in both CP/HCU (*p* = 0.041) and CP/LCU (*p* = 0.008) compared to TDs. All results remained unchanged after controlling for anxiety, depression and combined ADHD scores.

**Table 3:** Between group differences in brain activation across conditions.

	Mean (SD)			
Region	TD	CP/LCU	CP/HCU	ANOVA
ACC/mPFC	0.61 (0.577)	0.40 (0.715)	0.43 (0.762)	*F*(2, 92) = 0.85, *p* = 0.432
Insula	0.43 (0.349)	0.37 (0.607)	0.23 (0.549)	*F*(2, 92) = 1.28, *p* = 0.284
Occipital	0.57 (0.458)	0.16 (0.542)	0.23 (0.588)	*F*(2, 92) = 5.36, *p* = 0.006⁎

⁎ Significant at *p* < 0.05.

#### 3.3.3 Bayes-factor

To quantify the degree of certainty with which we can accept our findings, and in particular our null findings, we assessed the Bayes-factor for each of these comparisons (Table 4). This indicated that when comparing TDs and CP/HCU, there was only anecdotal support for the null hypothesis (i.e. no group differences in activation) in the AI (BF_10_ = 0.90), and ACC/mPFC (BF_10_ = 0.41), and substantial support for rejecting the null hypothesis in the occipital lobe (BF_10_ = 3.63). When comparing TDs and CP/LCU there was substantial evidence for the null hypothesis in the AI (BF_10_ = 0.28), only anecdotal support for the null hypothesis in the ACC/mPFC (BF_10_ = 0.51), and strong evidence for rejecting the null hypothesis in the occipital lobe (BF_10_ = 19.08).

**Table 4:** Bayes factor analysis for differences in brain regions.

	Bayes factor (BF_10_)	
Region	TD vs CP/LCU	TD vs CP/HCU
ACC/mPFC	0.51	0.41
Insula	0.28	0.90
Occipital	19.08	3.63

#### 3.3.4 Controlling for other psychiatric symptoms

As both CP/HCU and CP/LCU groups had elevated anxiety, depression and ADHD symptoms compared with the TD group and these are known to impact affective processing in fear inducing situations, we conducted post-hoc analyses on the Bayes factor estimates whilst controlling for anxiety, depression and ADHD total scores. This did not lead to any substantive differences in Bayes Factor estimates (i.e. no changes from or to ≥ substantial evidence for the null or alternate hypothesis).

#### 3.3.5 Post-hoc exploratory analyses

In order to further understand our findings, we conducted an additional set of post-hoc analyses to check whether the lack of robust activation of a core affective introspective network in the conjunction analysis for the CP groups was related to reduced activity in any particular condition. To this end we performed fear vs neutral analyses within each region for each CP group and for each condition separately (Table 5). As expected on the basis of the conjunction analysis, the TD group showed greater responsivity to fear than neutral stimuli across conditions and regions (all *p <* 0.002).

**Table 5:** Within group comparisons examining fear-neutral regional differences in activity within each condition.

		TD			LCU			HCU		
Condition	Region	Fear	Neutral	*p*	Fear	Neutral	*p*	Fear	Neutral	*p*
Own emotion	ACC/mPFC	0.66 (0.456)	0.46 (0.396)	0.001⁎	0.57 (0.513)	0.55 (0.426)	0.814	0.64 (0.625)	0.46 (0.470)	0.035⁎
	Insula	0.48 (0.263)	0.34 (0.264)	0.001⁎	0.41 (0.308)	0.37 (0.281)	0.405	0.50 (0.383)	0.37 (0.377)	0.002⁎
	Occipital	0.51 (0.603)	0.34 (0.690)	0.001⁎	0.39 (0.780)	0.36 (0.802)	0.531	0.46 (0.853)	0.37 (0.881)	0.094
Other emotion	ACC/mPFC	0.71 (0.461)	0.53 (0.408)	0.001⁎	0.65 (0.477)	0.46 (0.510)	0.012⁎	0.54 (0.498)	0.38 (0.512)	0.020⁎
	Insula	0.51 (0.290)	0.37 (0.297)	<0.001⁎	0.52 (0.377)	0.31 (0.341)	0.012⁎	0.40 (0.437)	0.35 (0.413)	0.442
	Occipital	0.55 (0.630)	0.36 (0.675)	<0.001⁎	0.46 (0.510)	0.38 (0.767)	0.217	0.48 (0.858)	0.39 (0.941)	0.137
Feel for other	ACC/mPFC	0.68 (0.502)	0.46 (0.416)	<0.001⁎	0.65 (0.416)	0.46 (0.421)	0.001⁎	0.45 (0.593)	0.37 (0.574)	0.303
	Insula	0.51 (0.319)	0.36 (0.256)	<0.001⁎	0.41 (0.262)	0.29 (0.308)	0.007⁎	0.44 (0.435)	0.40 (0.428)	0.366
	Occipital	0.60 (0.648)	0.39 (0.636)	<0.001⁎	0.48 (0.764)	0.43 (0.797)	0.362	0.52 (0.887)	0.47 (0.880)	0.447

⁎ Significant at *p* < 0.05.

The CP/HCU group only showed consistent responsivity to fear vs neutral stimuli when introspecting about their ‘Own emotions’ in the ACC/mPFC (*p* = 0.035) and AI (*p* = 0.002), with a trend in the occipital lobe (*p* = 0.094). In the ‘Other emotion’ condition, CP/HCU did show significant ACC/mPFC response (*p* = 0.020), but not in the AI (*p* = 0.442) or occipital lobe (*p* = 0.137). In the ‘Feel for other’ condition, CP/HCU did not show any significant responses in any of regions identified by our prior conjunction analyses (ACC/mPFC: *p* = 0.303; AI: *p* = 0.366; Occipital lobe: *p* = 0.447).

The CP/LCU group did not significantly differ in their responsivity to fear vs neutral stimuli during ‘Own emotion’ condition in any region examined (ACC/mPFC: *p* = 0.814; AI: *p* = 0.405; Occipital lobe: *p* = 0.531). However, they showed normal differentiation between fear and neutral stimuli in the ‘Other emotion’ and ‘Feel for other’ conditions in the ACC/mPFC (Other: *p =* 0.012; Feel for other: *p* = 0.001) and AI (Other: *p =* 0.012; Feel for other: *p* = 0.007), but not the occipital lobe (Other: *p* = 0.217; Feel for other: *p* = 0.362). In spite of these within-group differences, a group by fear-neutral interaction was not found for any condition or region (all *p* > 0.05), except the occipital lobe in the ‘Feel for other’ condition (*p* = 0.037).

## 4 Discussion

Here we used a task with evocative photographs and short auditory vignettes to investigate the neural basis of empathy in TD boys, boys with CP/HCU, and boys with CP/LCU. The task involved participants: (i) introspecting about their own feelings, (ii) thinking about what another person is feeling, and (iii) introspecting about their own feelings in response to hearing about another person in a fear-inducing situation. We reasoned that those brain areas commonly activated by TD children during these computations would be particularly important for resonating with and understanding other people's emotions and using one's own affective state to guide responding to others.

Our findings indicate that TD children show reliable differentiation between fear and neutral scenarios, across the task conditions, in two brain areas commonly associated with empathy/affective resonance - the AI and ACC/mPFC. They also showed increased activation for fear situations across conditions in the occipital cortex. This pattern of neural activation was not seen in either group of children with CP. However, direct comparisons between TD boys and both CP/HCU and CP/LCU boys could not definitively reject or support the existence of group differences across conditions in areas related to empathic processing/affective resonance (namely the AI and ACC/mPFC). Between-group comparisons and Bayes factor analyses are suggestive of reduced occipital activity in both CP/LCU and CP/HCU compared to TD children in a cluster centred on the lingual gyrus.

Exploratory analyses suggested context-specific abnormalities in affective introspection in the CP groups. Specifically, CP/HCU did not show significantly different neural responses between fear and neutral stimuli in the ‘Feel for other’ and to a lesser extent ‘Other emotion’ conditions. This suggests that within this group the lack of consistent conjunction effects may predominantly reflect abnormalities in affective introspection for others, as well as ability to perspective take about/understand other people's emotional states. By contrast, differentiation between fear and neutral stimuli within the CP/LCU group appeared to be reduced primarily within the ‘Own emotion’ condition, perhaps reflecting lesser engagement with introspection about one's own affective states. However, interpretation of these exploratory analyses should be viewed with caution, especially as no significant group by condition interaction was observed. Future work may also benefit from examining how the different regions involved in affective introspection and empathic processing may be particularly critical for certain aspects of information processing in different clinical groups. For instance, in the ‘Other condition’ we observe reduced differentiation between fear and neutral in the CP/HCU group only in the AI. Whilst the AI and ACC are largely co-activated during a range of processes, prior work and theoretical accounts have proposed distinct roles for these regions, with the AI acting as an ‘input’ node integrating awareness of emotional and physical states, and the ACC as an ‘output’ node necessary for the selection and preparation of responses to these states (Medford and Critchley, 2010).

The failure to find strong support for a group difference between TD and CP boys, in particular between TD and CP/HCU children as reported in many previous studies (e.g. Lockwood et al., 2013; Marsh et al., 2013; Michalska et al., 2016; Yoder et al., 2016), may have arisen for a number of reasons. First, our paradigm used mild (and thus ethically feasible) emotionally evocative scenes and vignettes, rather than more explicit video stimuli of pain and active harm. The responses to these stimuli may have therefore been more variable and potentially dependent on own prior experiences, exposure or emotional vulnerabilities, which could have increased within-group variance and made it more challenging to detect between-group differences. The fact that covarying anxiety scores resulted in the emergence of anecdotal evidence for group difference in AI responding between CP/CHU and TD groups, suggests that this may in part explain the pattern of findings in our study. It is also worth noting that many (though not all, see Marsh et al., 2013) previous studies examining incidental processing of affective/empathy inducing stimuli did not require participants to actively introspect about their emotions. Previous research suggests that when explicitly engaging in affective processing, even individuals with very high levels of psychopathic features (relative to TD individuals) engage brain areas involved in introspection (e.g. the AI), but under such conditions group differences are reduced (Meffert et al., 2013). It is possible that whilst incidental aspects of empathic processing are altered in CP/HCU individuals, deliberately engaging in introspection about emotions is not affected to the same degree. However, whether this translates to typical feelings of empathy is an empirical question that warrants further attention. It is possible that whilst incidental aspects of empathic processing are altered in CP/HCU individuals, deliberately engaging in introspection about emotions is not affected to the same degree. However, whether this translates to typical feelings of empathy is an empirical question that warrants further attention.

We were surprised to find significant group differences in occipital response between boys with CP and TD boys for fear compared to neutral scenes. This suggests greater attentional focus on the scenarios in the TD boys and this could, in part, explain the less reliable fear-neutral differentiation across conditions seen in CP/HCU and CP/LCU groups – as evidenced by the failure of the conjunction analyses to reliably detect the network including AI, ACC, and occipital areas, which was active across the conditions in TD children.

The present findings highlight the importance of contrasting different paradigms in studying affective and empathic processing. As noted above, most studies observing neural hypo-reactivity to affective stimuli in CP/HCU have been incidental processing paradigms (e.g. Lockwood et al., 2013; Marsh et al., 2013; Michalska et al., 2016; Yoder et al., 2016). One adult study has indicated that neural hypo-reactivity during empathic processing may be normalised during deliberative introspection on such stimuli (Meffert et al., 2013). The exploratory post-hoc analyses of the current study indicated that even during conditions that involve introspection, the neural response to empathic stimuli may not be fully normalised in children with CP, perhaps in particular those with CP/HCU. However, further studies using more salient stimuli and both incidental and deliberative conditions are required to fully investigate this possibility. Such studies are essential to elaborate on the nature of affective processing in CP children with various levels of CU traits.

Several other limitations not addressed so far may should be noted when interpreting the current study. First, although the stimuli we employed were designed to represent ‘fearful scenarios’, we cannot say with certainty that these elicited fear as opposed to another negatively valenced emotion. It is also unlikely that such scenarios would elicit fear to the same extent as actually experiencing a fear inducing situation. Future studies could add physiological measures of arousal to verify salience of the stimuli beyond self-report, but these measures are still limited in permitting any direct inference about a specific emotional state. Second, within this study we used a median split approach when deriving groups based on CU scores. Although this is a common approach within the field, future studies may benefit from including groups with more separation in their level of CU traits. Third, our task is also novel and has not been used in the field before. However, the robust engagement of the predicted brain areas in typically developing children using this task suggests that it is successful in quantifying the neural correlates of affective introspection in this group.

Overall, this study demonstrated that a core affective introspective network underpins introspection of one's own and others' emotions in TD children. This network, including the ACC/mPFC, anterior AI and occipital cortex did not appear to be consistently activated in CP/HCU or CP/LCU children, but no definitive evidence for robust group differences between TD and CP children was observed. This may be due to the task used in the current study, which involved a paradigm that utilised deliberate introspection and relatively mild affective stimuli. Exploratory post-hoc analyses also suggested that the CP groups may activate this network inconsistently, depending on the particular condition and that the two CP groups may show some dissimilarities in their empathic processing profiles. However, additional studies are required to further investigate the precise ways in which empathic processing is affected in different groups of children with CP.

## Appendix A Supplementary materials

Supplementary material Image 1
