# Supplementary material for: An fMRI investigation of empathic processing in boys with conduct problems and varying levels of callous-unemotional traits
Source: Neuroimage Clin. 2018 Feb 28;18:298–304. doi: 10.1016/j.nicl.2018.01.027 (PMC5987797; doi:10.1016/j.nicl.2018.01.027)
Supplement: Supplementary file 1 — Supplementary material [file mmc1.pdf]

Supplementary Materials

To create the image set for this task, 48 fear and 48 neutral scenes were obtained from Shutterstock and other public sources. Each image consisted of a scene, for example a poorly-lit underpass with graffiti on the walls, or a person's silhouette against a window (fear), or a person walking down a street (neutral). In a subset of stimuli, one or more people were visible. However, images were selected such that faces were obscured or indistinct. No scenes depicting people holding objects that could be construed as weapons were included. Brief vignettes were generated to provide a narrative to accompany the images. Audio recordings of the vignettes were made in an anechoic chamber, narrated in a neutral tone by a female speaker.

On the basis of a pilot study conducted with N=16 males (aged 12.7-17.0, mean age 15.3), the 36 most evocative images were selected as the fear stimuli, and the 36 least evocative were selected as the neutral stimuli. The remaining stimuli were used to familiarise participants with the task before they entered the scanner. Of the 72 experimental scenarios, three sets of 12 fear scenarios and three sets of 12 neutral scenarios were generated. Sets were matched on stimuli ratings, duration of the audio narrative, and the presence and number of people in the scene.
